# High-level production of violacein by the newly isolated *Duganella violaceinigra* str. NI28 and its impact on *Staphylococcus aureus*

**DOI:** 10.1038/srep15598

**Published:** 2015-10-22

**Authors:** Seong Yeol Choi, Sooyeon Kim, Sungsoo Lyuck, Seung Bum Kim, Robert J. Mitchell

**Affiliations:** 1 School of Life Sciences, Department of Biological Sciences, Ulsan National Institute of Science and Technology, Ulsan, S. Korea; 2 School of Life Sciences, Chungnam National University, Daejeon, S. Korea

## Abstract

A violacein-producing bacterial strain was isolated and identified as a relative of *Duganella violaceinigra* YIM 31327 based upon phylogenetic analyses using the 16S rRNA, *gyrB* and *vioA* gene sequences and a fatty acid methyl ester (FAME) analysis. This new strain was designated *D. violaceinigra* str. NI28. Although these two strains appear related based upon these analyses, the new isolate was phenotypically different from the type strain as it grew 25% faster on nutrient media and produced 45-fold more violacein. When compared with several other violacein producing strains, including *Janthinobacterium lividum, D. violaceinigra* str. NI28 was the best violacein producer. For instance, the crude violacein yield with *D. violaceinigra* str. NI28 was 6.0 mg/OD at 24 hours, a value that was more than two-fold higher than all the other strains. Finally, the antibacterial activity of *D. violaceinigra* str. NI28 crude violacein was assayed using several multidrug resistant *Staphylococcus aureus*. Addition of 30 μM crude violacein led to a 96% loss in the initial *S. aureus* population while the minimum inhibitory concentration was 1.8 μM. Consequently, this novel isolate represents a phenotypic variant of *D. violaceinigra* capable of producing much greater quantities of crude violacein, an antibiotic effective against multidrug resistant *S. aureus*.

Violacein is a bisindole derived from the condensation of two molecules of tryptophan^1^. This compound is vibrant purple in color and is known to possess a broad biological activity, including performing as an antitumor^2^, antifungal^3^ and antiviral^4^. Although not tested within humans to date, violacein has been found to have no adverse impact on mice when administered at a concentration of 1 mg/kg^2^. It has recently garnered more attention, however, due to its antibacterial activity against *Staphylococcus aureus*^5^,^6^. Tests with this pathogen found that violacein is capable of both acting as a bacteriostatic and as a bacteriocidal agent against *S. aureus* depending on the concentration used^7^. Moreover, a recent study demonstrated that violacein works synergistically with other antibiotics, leading to significantly lower minimum inhibitory concentrations for *S. aureus*, *Klebsiella pneumonia* and *Pseudomonas aeruginosa*^6^. All three of these strains are included in the ESKAPE pathogen grouping, a list of multidrug resistant bacteria that are becoming more prominent within healthcare environments and nosocomial infections^8^,^9^. As such, research into the isolation and characterization of violacein producing bacteria, the cloning and heterogeneous expression of the *vioABCDE* genes and the fermentative production of violacein has recently blossomed with several key studies reported within the last couple of years^10^,^11^,^12^,^13^.

Violacein is produced by numerous bacterial strains spanning various genera, including *Chromobacter*^14^,^15^, *Pseudoalteromonas*^16^,^17^, *Janthinobacterium*^18^,^19^ and *Duganella*^20^. Moreover, violacein producing bacterial strains have been isolated from diverse environmental locales. *Janthinobacterium*^18^,^21^,^22^, for instance, was isolated from a glacier, *Chromobacteria* from a river^14^,^23^, *Duganella* from agricultural and forest soils^24^,^25^ and *Collimonas* from the sea^26^. In this study, we report on the isolation and initial characterization of a natural soil isolate, *Duganella violaceinigra* str. NI28, obtained from a temperate forest soil sample taken near Ulsan, South Korea. This strain produces violacein at much higher rate and levels than the type strain *D. violaceinigra* YIM 31327^24^,^27^.

## Results

### Isolation and Identification of *D. violaceinigra* str. NI28

**Figure 1:**
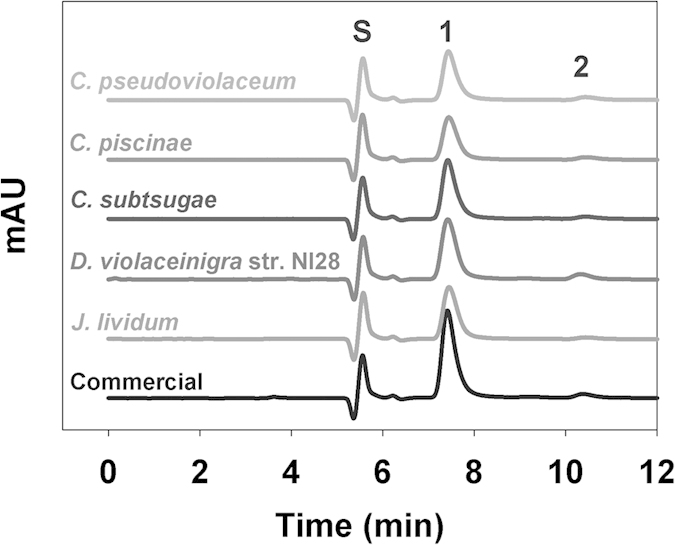
HPLC analysis of the violacein extracted from cultures of *D. violaceinigra* str. NI28 and several of the strains employed in this study, showing the presence of violacein (Peak #1–7.5 min) and a second peak (Peak #2–10.3 min) that is presumed to be deoxyviolacein. The solvent front (Peak S) is seen at 5.4 min. A plot generated using a commercially available violacein extracted from *Janthinobacterium lividum* is also provided for comparison and includes both Peak #1 and Peak #2.

Various natural bacterial isolates from a forest soil sample were grown on nutrient agar (NA) and a single colony that had a dark purple hue, suggesting that this strain produced the bisindole violacein, was selected for further characterization. Production of crude violacein by this strain was demonstrated using HPLC analysis (Fig. 1). When the crude violacein extracted from our new isolate was compared with a commercial preparation of crude violacein from *Janthinobacterium lividum* (Sigma-Aldrich, USA), they were basically indistinguishable from each other.

**Figure 2:**
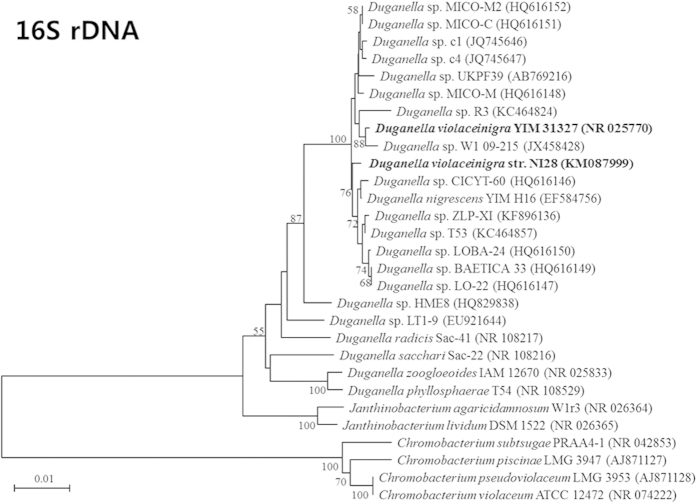
Phylogenetic tree using the 16S rRNA gene sequence. The numbers at nodes indicate levels of bootstrap support (%) based on 1,000 resampled dataset. The bars corresponds to 0.01 or 0.05 substitutions per nucleotide. In parentheses are the nucleotide sequence accession numbers of corresponding strains.

**Figure 3:**
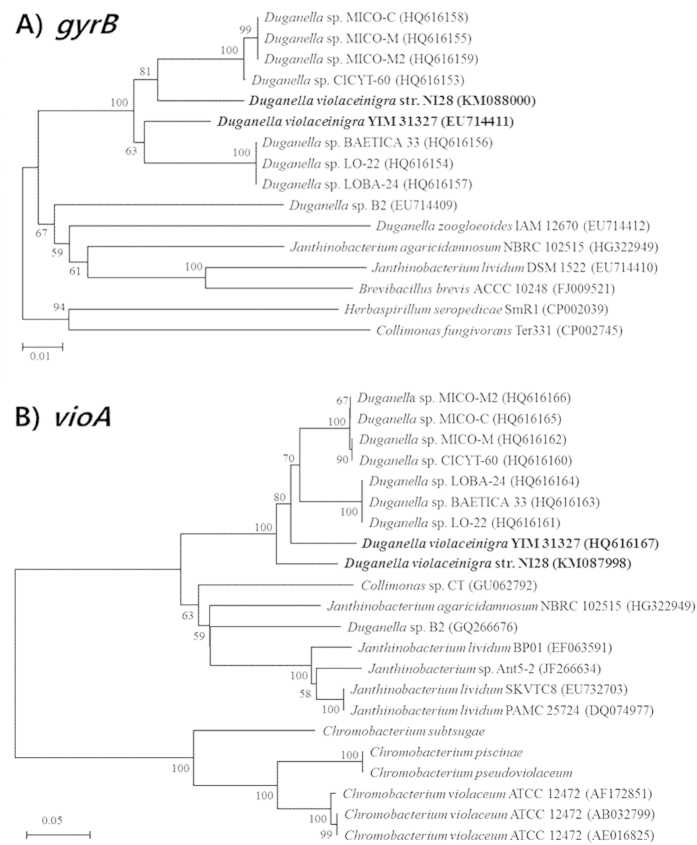
Phylogenetic analysis of the new isolate using the (A) *gyrB* and (B) *vioA* gene sequences. The numbers at nodes indicate levels of bootstrap support (%) based on 1,000 resampled dataset. The bars corresponds to 0.01 or 0.05 substitutions per nucleotide. The nucleotide sequence accession numbers of the corresponding strains are listed in theparentheses.

Identification of this strain was next performed using the sequences obtained from three gene loci (16S rRNA, *gyrB* and *vioA*), with each confirming that it was closely related to *Duganella violaceinigra* YIM 31327 (Figs 2 and 3). The level of sequence similarities between NI28 and *D. violaceinigra* YIM 31327 were 98.8% for 16S rRNA gene, 95.9% for *gyrB*, and 88.8% for *vioA*, thus indicating that NI28 is close, but not identical to *Duganella violaceinigra* YIM 31327. This relationship was further supported by a fatty acid methyl ester (FAME) analysis and comparison of this strain and the type strain, *Duganella violaceinigra* YIM 31327, which showed only minor differences between the two strains (Table 1). Based upon the phylogenetic and FAME analyses, this strain was designated *D. violaceinigra* str. NI28 and deposited within the Korea Agricultural Culture Collection (www.genebank.go.kr) (KACC 91951P).

### *D. violaceinigra* str. NI28 is Phenotypically Variant from *D. violaceinigra* YIM 31327

**Figure 4:**
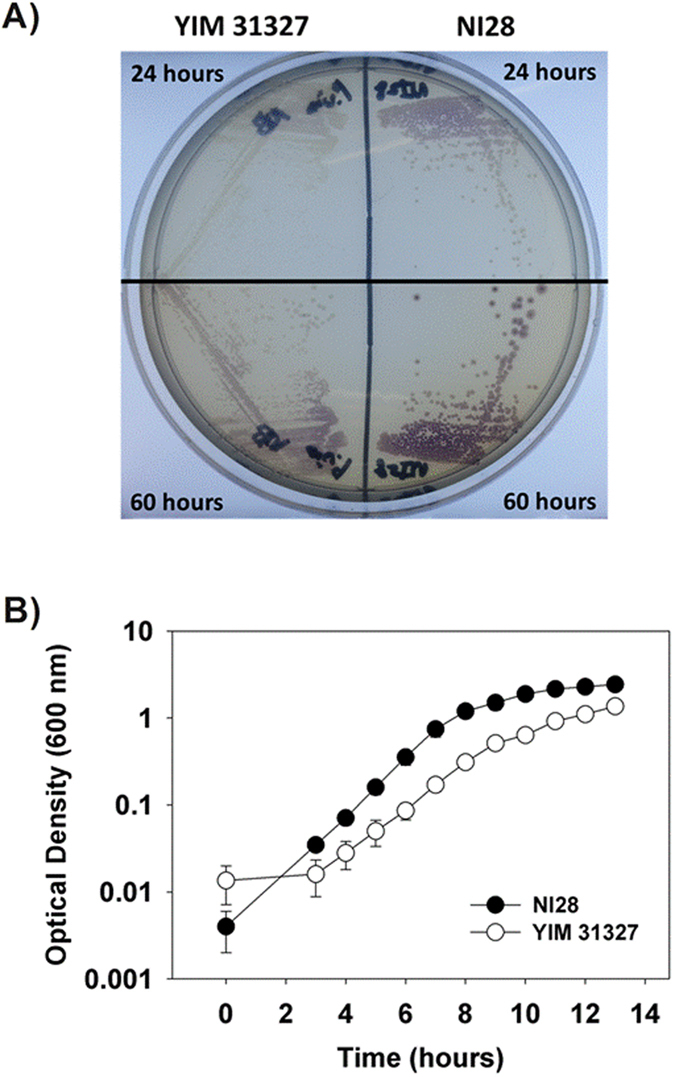
*D. violaceinigra* str. NI28 grows faster than *D. violaceinigra* YIM 31327. (**A**) Image of each strain grown on NB agar plates for 24 and 60 hours. The upper and bottom regions are from the same plate. Note the more rapid colony development and violacein production by *D. violaceinigra* str. NI28. (**B**) Growth of both strains in NB media confirming that *D. violaceinigra* str. NI28 grows faster. The data for each strain was obtained from three independent cultures.

Although both *D. violaceinigra* str. NI28 and *D. violaceinigra* YIM 31327 were isolated from forest soils and are related genetically based upon the above results, differences were readily apparent between them. For instance, *D. violaceinigra* str. NI28 was found to have trypsin activity using the API ZYM Kit (bioMerieux, France) while *D. violaceinigra* YIM 31327 was negative for this protease. Furthermore, *D. violaceinigra* str. NI28 grew remarkably well and produced a significant amount of violacein when cultured on NA (Fig. 4A). This was in stark contrast with *D. violaceinigra* YIM 31327, which grew slower and was much less proficient at producing violacein. Figure 4A shows that colonies of *D. violaceinigra* str. NI28 were larger and already producing violacein after 24 hours of growth while those of *D. violaceinigra* YIM 31327 were smaller and still pasty in color. Only after 60 hours did the *D. violaceinigra* YIM 31327 colonies achieve a similar size and hue as 24 hour-old the *D. violaceinigra* str. NI28 colonies (Fig. 4A).

**Figure 5:**
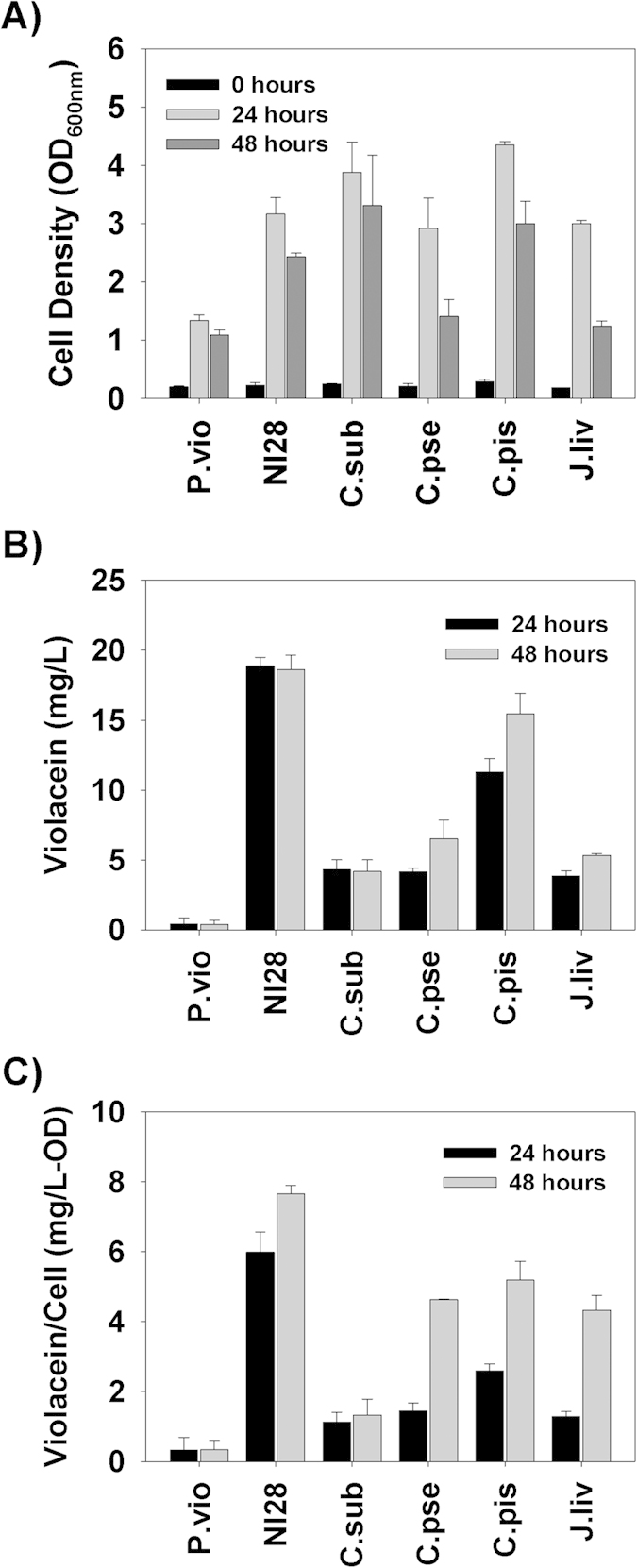
Growth and violacein production by several different bacterial strains. (**A**) Optical density values for each culture at 0, 24 and 48 hours. (**B**) Violacein concentration within each culture at 24 and 48 hours, showing the rapid and high level violacein generation by *D. violaceinigra* str. NI28 as compared with the other strains. (**C**) The relative concentration of violacein based upon the culture density, illustrating that *D. violaceinigra* str. NI28 gave the greatest yields.

Both of these findings were further evidenced in liquid cultures. As with the colonies, *D. violaceinigra* YIM 31327 was slower to grow in NB liquid media (Fig. 4B). The doubling time for *D. violaceinigra* YIM 31327 was 71 minutes based upon the logarithmic growth stage between 3 and 7 hours. In contrast, *D. violaceinigra* str. NI28 doubled every 53.7 minutes, a value that is 25% faster than *D. violaceinigra* YIM 31327. Not only did the newly isolated *D. violaceinigra* str. NI28 grow faster but the optical density after 24 hours was significantly higher (2.4-fold), as shown in Figs 4B and 5A. We also noticed that *D. violaceinigra* str. NI28 tended to form flocs when grown in liquid cultures while *D. violaceinigra* YIM 31327 cells generally remained suspended (data not shown). It is not clear what benefit *D. violaceinigra* str. NI28 gains from forming these aggregates, but it was reported that flocs may help protect against predation^28^.

### *D. violaceinigra* str. NI28 is a Prolific Violacein Producer

All three of these growth characteristics (rate, yield and floc formation) may contribute to the higher violacein production seen with this new strain. As shown in Fig. 5B, *D. violaceinigra* str. NI28 was a much more proficient violacein producer than *D. violaceinigra* YIM 31327 under these conditions, generating as much as 18.9 mg/L of crude violacein after 24 hours. Further incubation for an additional 24 hours did not improve upon this yield with 18.6 mg/L from the two day culture. *D. violaceinigra* YIM 31327, on the other hand, produced only 0.42 mg/L during the first 24 hours, and this dropped to 0.38 mg/L after 48 hours. Considering both time points, the new strain produced approximately 45-fold more violacein than the type strain.

Although *D. violaceinigra* str. NI28 is clearly better than *D. violaceinigra* YIM 31327 at producing violacein, the literature also lists several other violacein producing strains, including *Chromobacter* species and *Janthinobacterium lividum* (Table 2). Consequently, we cultivated each of these strains in NB media and determined both the optical density (OD) and crude violacein yields after 24 and 48 hours (Fig. 5). The 24 and 48 hour *D. violaceinigra* str. NI28 optical densities were similar with most of the other violacein producing strains but both *C. piscinae* and *C. subtsugae* grew to higher ODs (Fig. 5A). However, of all the strains tested, the new isolate gave the highest crude violacein yields, particularly when grown for 24 hours (Fig. 5B). This figure also shows that it was one of only two strains, other than *D. violaceinigra* YIM 31327, which reached a maximum violacein concentration in 24 hours. This rapid and high level production by *D. violaceinigra* str. NI28 in NB clearly sets this strain apart from the other strains, particularly its closest relative, *D. violaceinigra* YIM 31327, which was the poorest violacein producer tested in this study.

When the quantity of violacein produced by all six strains was normalized using the OD, *i.e.*, the specific productivity, the newly isolated *D. violaceinigra* str. NI28 gave the best results (Fig. 5C). This was particularly true for the 24 hour sample where the specific productivity was 6.0 mg crude violacein/OD, a value that was more than twice that obtained for the next closest strain, *C. piscinae* (2.6 mg crude violacein/OD). In comparison, the specific productivity from the type strain, *D. violaceinigra* YIM 31327, was the lowest of all the strains tested (0.33 and 0.34 mg/OD at 24 and 48 hours, respectively). As such, *D. violaceinigra* str. NI28’s high level production of violacein implies that this strain contains more violacein per cell than the other strains, a finding which may contribute to making downstream purification of violacein easier.

### *Duganella violaceinigra* str. NI28 Violacein is Bacteriocidal Towards *Staphylococcus aureus*

**Figure 6:**
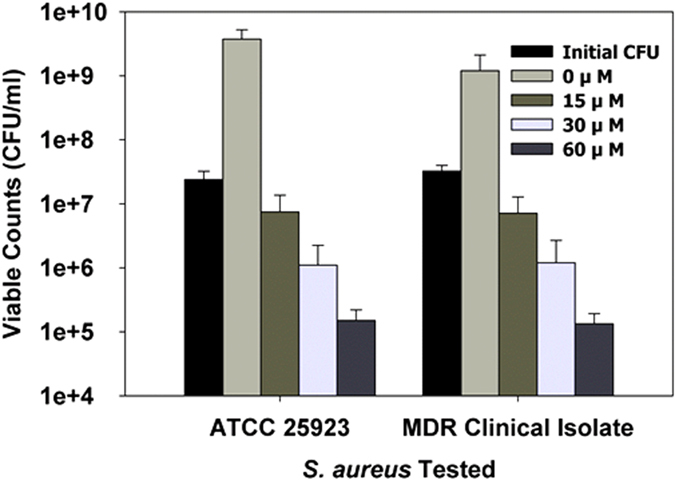
Inhibition and killing of *Staphylococcus aureus* by *D. violaceinigra* str. NI28 violacein. Two *S. aureus* strains were tested: ATCC 25923 and a clinical isolate. The initial CFU was determined just prior to addition of the violacein. The viability of the other samples was determined after a 24 hour exposure to the violacein at the concentrations listed, showing that the addition of 15 μM or greater violacein was bactericidal towards both pathogenic strains. The samples were prepared so that each had the same amount of DMSO (1.3%) added. The concentrations listed are based upon the commercial standard.

Several recent articles highlighted the activity of violacein against the human pathogen *Staphylococcus aureus* and showed that a crude violacein concentration of 17 μM was capable of inhibiting growth of this strain^5^,^6^. Similarly, we tested the activity of *D. violaceinigra* str. NI28 violacein extracts with two *S. aureus* isolates that were obtained from culture repositories (ATCC 25923 and the CCARM strains) and from a patient infected with a multidrug-resistant *S. aureus* (Clinical) (Table 3). As shown in Fig. 6, the crude violacein prepared from *D. violaceinigra* str. NI28 was capable of blocking growth of both *S. aureus* strains when added to a final concentration of 15 μM, or approximately 5 mg/L. Moreover, this concentration led to a 70% loss in viability after 24 hours when compared with the initial *S. aureus* colony-forming unit (CFU) values while higher concentrations led to comparably greater losses in viability. For instance, 60 μM violacein reduced both *S. aureus* populations by an average of 99.5% when compared with the initial CFU numbers.

We next tested the efficacy of the *D. violaceinigra* str. NI28 crude violacein against a selection of bacterial strains, including several multidrug resistant *S. aureus* strains that have diverse antibacterial resistance footprints according to the EUCAST breakpoints (Table 3). Table 4 lists the minimal inhibitory concentrations (MICs) of violacein found for each of the strains tested in this study. Growth of all *S. aureus* cultures was inhibited by a violacein concentration of 15 μM (Table 4) regardless of the antibiotic resistant nature of the pathogen. Furthermore, MIC tests performed using *Pseudomonas aeruginosa* and *Klebsiella pneumoniae* found both of these pathogens were resistant to violacein. Parallel tests performed with a commercially available crude violacein from *J. lividum* gave identical results (Table 4), demonstrating that the bioactivity of the crude violacein from *D. violaceinigra* str. NI28 against these pathogens is equivalent to that of the commercially available *J. lividum* crude violacein.

## Discussion

As a bacterial secondary metabolite, violacein has garnered renewed interest lately. A vibrant violet-hued bisindole derivative of tryptophan, violacein has been shown to have multiple biological activities, including as an anticancer^2^,^13^ and antibiotic, particularly against *S. aureus*. It is this multifaceted character of violacein, and the fact that resistance to this antibiotic has not yet been described, that makes it an attractive product for bioprocesses.

This study reports on the isolation and characterization of a novel high-level violacein-producing bacterial strain. This strain was identified as *Duganella* using both FAME and phylogenetic analyses and found to be similar based upon these tests with the type strain *D. violaceinigra* YIM 31327, which also produces violacein. However, when these two strains were compared with each other, we found that they were quite distinct from each other. For instance, the new strain grew faster and produced significantly more violacein (45-fold) than *D. violaceinigra* YIM 31327. Moreover, this new isolate also has a tendency to form flocs, another attribute that has been noted as being more economical when harvesting microbial biomass^29^. When this strain, *D. violaceinigra* str. NI28, was compared with several other well-known violacein producers, it grew faster, produced more violacein and had the best yields per biomass. Both the rapid, high-level production by *D. violaceinigra* str. NI28 and its higher concentration within the cells are clearly advantageous and desirable from an economic viewpoint since the first would reduce the time needed for production of violacein while the second minimizes the biomass needed for its subsequent extraction and purification. These characteristics of *D. violaceinigra* str. NI28 make it a potentially attractive and alternative strain for the commercial production of violacein.

When the violacein from *D. violaceinigra* str. NI28 cultures was purified and analyzed, we found that it was similar with a commercially available preparation from *J. lividum*, as well as that extracted from the various strains employed in this study. As noted above, this bisindole is quite effective against *S. aureus* and, as such, this pathogen was selected for this study due to its overwhelming presence within nosocomial infections^30^,^31^ and its broad resistance to antibiotics^32^. Previous studies reported the minimum inhibitory concentration (MIC) of violacein was 5.7 μg/ml, or approximately 17 μM, with *S. aureus*^6^. The preparation from *D. violaceinigra* str. NI28 was equally potent with an MIC of 5 μg/ml, or approximately 15 μM. This concentration was not only inhibitory but also bactericidal as it led to a 69% reduction in the viability with the ATCC strain and a 78% decrease with the clinical isolate. Both of these findings clearly illustrate that the crude violacein purified from cultures of *D. violaceinigra* str. NI28 is capable of not only inhibiting *S. aureus* growth but also killing this pathogen.

Moreover, the multidrug resistant nature of the *S. aureus* strains did not contribute to their survival when exposed to violacein. Tests using a total of five *S. aureus* strains with different antibiotic resistance footprints, showing that several were multidrug resistant (Table 3), found that each was equally sensitive to the crude violacein from *D. violaceinigra* str. NI28, with MICs of 15 μM (Table 4). These values are basically identical with that reported by Subramaniam *et al.* (2014), where they found the MIC for *S. aureus* to be 5.7 μg/ml (17 μM). When we performed further MIC tests in a modified M9 media, the effective concentration dropped to only 0.9 or 1.8 μM, a value that is significantly lower than those found with the Mueller-Hinton media and is likely attributable to the nutrient poor nature of the medium used. Similar impacts of the media richness and bacterial responses has been documented previously^33^, where the authors found the use of a nutrient poor medium made the bacteria more susceptible to chemicals. Consequently, the use of a minimal media within this study increased the susceptibility of the bacteria to violacein and does not indicate a greater activity against these pathogens. Further evidence of this is seen in the similar MICs obtained with the commercially available *J. lividum* crude violacein, which was also significantly lower (Table 4). The HPLC analyses of these two crude violacein preparations, shown in Fig. 1, found them to be similar. The second peak was not identified but is presumed to be deoxyviolacein since this compound is also known to be generated alongside violacein. Although it may also play a role and contribute to *S. aureus* inhibition, a recent study found that deoxyviolacein was less effective against this pathogen than violacein^34^.

Two additional pathogens were also tested, *P. aeruginosa* PAO1 and *K. pneumoniae*, but neither of these was sensitive to either of the violacein samples (Table 4). This was not unexpected as several articles previously highlighted the tendency for Gram-positive strains to be more sensitive than Gram-negative strains^6^,^25^. These findings suggest that the presence of the outer membrane within these bacteria provides protection against violacein and its activity and is an area for further evaluation in a later study.

In conclusion, a violacein-producing strain was isolated from a forest soil sample in Ulsan, South Korea. This strain, *D. violaceinigra* str. NI28, produces violacein at quicker and at higher levels than other comparable strains, particularly the type strain *D. violaceinigra* YIM 31327, when grown under the conditions used in this study. Although the violacein concentrations produced in this study are low, a group recently reported achieving as much as 0.82 g/L with *C. violaceum* by optimizing the conditions for fermentation and the media used^35^. As *D. violaceinigra* str. NI28 is a new strain and, based upon the violacein production and yields found in this study, is generally equivalent to or better than the other strains tested, expanding its growth and violacein production through similar techniques would likely increase the yields. Moreover, other recent studies have shown the successful expression of the *vioABCDE* operon within other bacterial hosts to produce even higher levels of violacein. The characteristics of *D. violaceinigra* str. NI28 make its *vioABCDE* operon an interesting addition to these studies and, as such, cloning of these genes for their heterologous expression may likewise be beneficial.

## Methods

### Isolation of *Duganella violaceinigra* str. NI28

The novel strain characterized in this study, *i.e.*, *Duganella violaceinigra* str. NI28, was isolated using soil obtained from a temperate forest nearby the campus. For this, a 50 g sample of soil was mixed with 500 ml of sterile tap water and shaken at 30 °C for 2 hours. Afterwards, the soil particulates were centrifuged at 500 × g for 5 minutes and the supernatant sampled. Aliquots (100 μl) of this solution were spread out on nutrient agar (NA) (1.6% agar; Difco, USA) plates. A total of forty plates were prepared and these were then incubated at 30 °C for 48 hours. A single colony showing a violet hue on one of the plates was selected for further evaluation.

### Bacterial Strains and Growth

The bacterial strains used in this study are listed in Table 2. All of the strains were grown from −80 °C freezer stocks that were prepared individually. For the violacein producing strains, the stocks were prepared by adding DMSO (Sigma-Aldrich, USA) to a final concentration of 10%. The *Staphylococcus aureus* strains were stored using glycerol (Sigma-Aldrich, USA) at a final concentration of 25%.

Each violacein producing strain was struck out on NA plates and grown at 30 °C overnight. A single colony was transferred to a sterile 15 ml conical tube (SPL, Korea) containing 3 ml of nutrient broth (Difco, USA). This culture was grown at 30 °C and 250 rpm overnight. To 20 ml of fresh NB medium in a 100 ml baffled flask to increase aeration, 2 ml (10% v/v) of the overnight culture was added and this was again shaken at 30 °C and 250 rpm for 48 hours. At set times during this cultivation (24 and 48 hours), aliquots were taken to determine the culture optical density and violacein concentration as described below. For the growth curves shown in Fig. 4, 100 ml cultures were cultivated within 250 ml flasks. All the other strains were grown using the same protocols except at 37 °C and in TSB media (Difco, USA). Other media was used for the antibiotic resistance characterization and minimum inhibitory concentration determination as described below.

### Identification of *D. violaceinigra* str. NI28 by 16S using phylogenetic markers

The genus of isolate NI28 was identified using three genes, namely 16S rRNA, *gyrB* and *vioA*. The primers for PCR amplification of the genes were 27f (5′-AGA GTT TGA TCM TGG CTC AG-3′) and 1492r (5′-GGT TAC CTT GTT ACG ACT T-3′) for the 16S rRNA gene, Up-1G (5′-YGC SGG CGG YAA GTT CGA-3′) and Up-2G (5′-CCR TCG ACG TCV GCR TCG GT-3′) for *gyrB*^36^, and VPA3 (5′-CCR CAG CTS CAY CCG CAT TTC CAG-3′) and VPA4 (5′-CAG GCY GCC CTC CAT CCA GCC RCA-3′) for *vioA*^26^. The PCR conditions for the *gyrB* gene were: 95 °C for 5 minutes followed by 35 cycles of 95 °C for 30 s, 60 °C for 15 s and 72 °C for 90 s. The final extension was at 72 °C for 5 minutes. The *vioA* gene was amplified in the same manner but with an annealing temperature of 62 ºC. The phylogenetic analysis between NI28 and the related taxa including *Duganella* was performed using MEGA 6.0^37^. Neighbor-joining trees were inferred based on Jukes-Cantor evolutionary distances for each gene. The trees were evaluated using bootstrap analysis with 1,000 resampled datasets. The sequences of each gene from strain NI28 were deposited within the GenBank database under the accession numbers (KM087998, KM087999 and KM088000).

### Fatty Acid Methyl Ester Preparation and Analysis

Colonies of *D. violaceinigra* str. NI28 and *D. violaceinigra* YIM 31327 grown on NA plates were used for this analysis. To prepare the samples, several colonies were transferred to a test tube and 1 ml of Reagent #1 (45 g NaOH (ACS Grade, Sigma-Aldrich, USA), 150 ml HPLC Grade methanol (Sigma-Aldrich, USA) and 150 ml deionzed distilled water) was added. After vortexing to suspend the bacteria, this sample was placed at 100 °C for 5 minutes, vortexed once more and then incubated at 100 °C once more for 25 minutes. Afterwards, the tubes and samples were cooled down in a room temperature water bath. To initiate methylation of the fatty acids, 2 ml of Reagent #2 (325 ml 6 N HCl (Sigma-Aldrich, USA) mixed with 275 ml HPLC Grade methanol (Sigma-Aldrich, USA)) was added to each tube, which was vortexed and then incubated at 80 °C for 10 minutes. Afterwards, the tubes were quickly cooled by gently shaking them in a room temperature water bath. To each tube, 1.25 ml Reagent #3 (prepared using an equal volume of HPLC Grade hexane (Sigma-Aldrich, USA) and HPLC Grade methyl tert-butyl ether (Sigma-Aldrich, USA)) was added and the contents were gently mixed end-over-end for 10 minutes using a rotator. The aqueous phase was removed from the tubes and discarded. To remove any free fatty acids, the samples were washed with 3 ml of Reagent #4 (10.8 g ACS Grade sodium hydroxide in 900 ml deionized distilled water) by gently mixing them as above for 5 minutes. Once more, the aqueous phase was removed and discarded. The organic phase was used for gas chromatography (GC) analysis. For the GC analysis, the column was an Ultra 2 (25 m, 0.2 mm, Agilent, USA). The protocol used was the same as used previously for the *D. violaceinigra* type strain^24^.

### Phenotypic Assessment of *D. violaceinigra* str. NI28

A comparative phenotypic analysis of *D. violaceinigra* str. NI28 and YIM 31327 was performed using the API 20NE and API ZYM test kits (bioMerieux, France) according to the manufacturer’s suggested protocol.

### Extraction and HPLC Analysis of the Violacein Produced by *D. violaceinigra* str. NI28

After growth for 24 and 48 hours, 1 ml of the culture was pelleted by centrifugation for one minute at 16,200 × g and room temperature. After aspirating off the media, the pellet was resuspended in an equal volume of ethanol and shaken at 37 °C and 250 rpm for 24 hours. The solution was then transferred to another centrifuge tube and the absorbance measured at 575 nm. This value was used to calculate the violacein concentration using an extinction coefficient of 73.4 L mg^−1^ cm^−1 12^. The crude violacein concentration within the ethanol extracts was determined using standards prepared with a crude violacein purchased from Sigma-Aldrich (USA, Cat. No. V9389).

The crude violacein sample was also analyzed using HPLC 1200 (Agilent, USA) as described previously^12^. For this, 10 μl samples of the ethanol extracted violacein were analyzed using a C-18 column (Hypersil ODS, 5 μm, 250 × 4.6 mm) at 30 ºC. The mobile phase was 50% denatured ethanol (HPLC Grade, Sigma-Aldrich, USA) and detection was performed at 575 nm using an Agilent 1260 Infinity ELSD. The violacein concentration within the ethanol extracts was determined as described above using crude violacein purchased from Sigma-Aldrich (USA, Cat. No. V9389) as a known standard. For the viability and MIC tests, the ethanol was dried off and the crude violacein powder solubilized within DMSO at a known concentration.

### Antibiotic Disc Diffusion Assay

Each of the strains characterized is listed in Table 3. Each strain was grown on tryptone soy agar plates overnight and several colonies were transferred to 5 ml fresh sterile tryptone soy broth (TSB). They were then grown for overnight (250 rpm, 37 ºC), diluted into fresh sterile TSB (0.1%) and grown until the optical density (OD) was equivalent to a 0.5 McFarland standard, which was provided with the API tests used above. After reaching this OD, the culture was spread out on Mueller-Hinton agar plates using sterile, non-toxic cotton swabs. The plates were air-dried for 10 minutes before applying the antibiotic discs (BBL Sensi-Disc, BD, U.S.A.). The antibiotics tested were ciprofloxacin, clindamycin, erythromycin, gentamycin, oxacillin, rifampin, tobramycin and vancomycin. The zones of growth inhibition were determined after 18 hours of incubation at 37 °C for all the antibiotics except vancomycin and oxacillin, which were determined after 24 hours. Strain resistance was determined using the latest breakpoint tables available at the European Committee on Antimicrobial Susceptibility Testing (EUCAST) website (http://www.eucast.org/clinical_breakpoints/).

### Violacein Killing of *Staphylococcus aureus*

The extracted crude violacein was concentrated by drying down multiple preparations under nitrogen gas and resuspending it in DMSO. The concentration was once more determined as described above.

Two different strains of *S. aureus* (ATCC 25923 and a clinical isolate) were used to evaluate the activity of the violacein extracted from *P.* sp. NI28 cultures. After growth overnight, the *S. aureus* cultures were diluted 1:100 into 3 ml M9 modified minimal media containing 1 g/L tryptone and 0.2% glucose. We chose this media as it is slightly modified from one that was published recently and used for MIC testing^38^. Violacein (M.W. 343.33) in DMSO was added to these cultures at a final concentration of 0, 15, 30 or 60 μM. The final DMSO concentration in all cultures was 1.3%. The cultures were then incubated at 37 °C and 250 rpm for 24 hours, after which the viable *S. aureus* numbers were determined using serial dilutions of the cultures that were spread out on TSB agar plates and grown overnight at 37 ºC.

### Minimum Inhibitory Concentration Determination

The crude violacein samples were prepared as above and the commercial violacein was purchased from Sigma-Aldrich (USA). Determining the minimum inhibitory concentration was performed as described previously using resazurin^39^, with some modification. Briefly, the bacteria were grown overnight in cation adjusted Mueller-Hinton broth (10 mg/L of both Ca^2+^ Mg^2+^) medium at 37 °C and 250 rpm and then sub-cultured into fresh media and grown to an OD_600nm_ of 0.1 under the same conditions. When they reached this OD, the cultures were diluted 1:100 into either cation adjusted Mueller-Hinton broth or modified M9 minimal media (containing 1 g/L tryptone and 0.2% glucose).

The media for the plate was prepared separately. Briefly, 3 ml of a sterile 0.2% resazurin solution prepared using distilled water was diluted into 12 ml of the modified M9 media. A 1.5 ml sample of this was then taken and crude violacein was added to a final concentration of 60 μM. Within the first row of the plate (row A), 200 μl of either cation adjusted Mueller-Hinton broth or modified M9 media containing 60 μM violacein was added. Each of the other wells had 100 μl of the same media containing resazurin added to them. The violacein containing media was then diluted two-fold sequentially from rows A-G but not into the final row (H). To the wells, 100 μl of the prepared bacterial cultures were added, giving a final cell concentration of 4 × 10^5^ CFU/well, the highest violacein concentration of 30 μM and a resazurin concentration of 0.02% in each well. The final row (H) was used as a positive control to demonstrate that each bacterium was capable of growing. Each strain was tested independently in triplicate. The control wells had only the media and the violacein added to them to test for contamination. All the wells had a final DMSO concentration of 1%.

### Data Analysis

Each of the experiments was performed in triplicate for error analysis. The standard deviations are presented as error bars in each graph where appropriate.

## Additional Information

**How to cite this article**: Choi, S.Y. *et al.* High-level production of violacein by the newly isolated *Duganella violaceinigra* str. NI28 and its impact on *Staphylococcus aureus*. *Sci. Rep.* **5**, 15598; doi: 10.1038/srep15598 (2015).

## Figures and Tables

**Table 1:** Fatty acid methyl ester (FAME) comparison between *D. violaceinigra* str. NI28 and *D. violaceinigra* YIM 31327.

Fatty Acid (%)	NI28	YIM 31327
Straight Chain		
C_10:0_	−	0.4
C_12:0_	5.05	6.54
C_14:0_	2.99	1.04
C_16:0_	33.28	31.42
Branched		
Iso-C_10:0_	0.12	−
Unsaturated		
C_14:1_	−	0.26
C_18:1_	3.8	3.69
Hydroxy		
C_10:0_	−	3.82
C_12:0_	2.66	1.74
C_14:0_	3.41	3.6
Sum in Feature 3	48.68	47.49

*Summed features represent two or three fatty acids that cannot be separated by the Microbial Identification System. The fatty acids included in Feature 3 consist of C_16 :1_ω7c and/or iso-C_15:0_ 2-OH.

**Table 2:** Strains used in this study.

Strains	Description	Reference^a^
*Duganella violaceinigra* str. NI28	Violacein producer	This study
*Duganella violaceinigra*	Violacein producer	YIM 31327
*Janthinobacterium lividum*	Violacein producer	ATCC 12473
*Chromobacterium pseudoviolaceum*	Violacein producer	ATCC 7461
*Chromobacterium piscinae*	Violacein producer	LMG 3947
*Chromobacterium subtsugae*	Violacein producer	DSM 17043
*Staphylococcus aureus*	Type strain	ATCC 25923
*Staphylococcus aureus*	Multidrug Resistant (MRSA)	Clinical Isolate
*Staphylococcus aureus*	−	CCARM 0201
*Staphylococcus aureus*	Multidrug Resistant (MRSA)	CCARM 3090
*Staphylococcus aureus*	Multidrug Resistant (MRSA)	CCARM 3840
*Klebsiella pneumoniae*	Human pathogen	ATCC 13883
*Pseudomonas aeruginosa* PAO1	Human pathogen	

^a^YIM (Yunnan Institute of Microorganisms); ATCC (American Type Culture Collection); LMG (Belgian Coordinated Collection of Microorganisms); DSM (Deutsche Sammlung von Mikroorganismen und Zellkulturen GmbH); CCARM (Culture Collection of Antimicrobial Resistant Microbes).

**Table 3:** 

Strain	Van^a^	Rif^a^	Cip^a^	Clin^a^	Oxa^a^	Ery^a^	Gent^a^	Tob^a^
*S. aureus*								
ATCC 25923	19–21(S)^b^	32–36(S)	30–31(S)	27–30(S)	22–25(S)	27–30(S)	24–27(S)	26–27(S)
Clinical Isolate	21–22(S)	**0(R)**	**11**–**18(R)**	**0(R)**	**0(R)**	**0(R)**	**0(R)**	**0(R)**
CCARM 0201	18–19(S)	31–34(S)	25–30(S)	23–28(S)	25–27(S)	21–30(S)	22–24(S)	20–23(S)
CCARM 3090	19–21(S)	36–38(S)	**0(R)**	**0(R)**	**0(R)**	**0(R)**	26–27(S)	26–28(S)
CCARM 3840	19–24(S)	33–40(S)	**0(R)**	**0(R)**	**0(R)**	**0(R)**	**0(R)**	**0(R)**
*K. pneumoniae* ATCC 13883	ND^c^	ND	35–37(S)	ND	ND	ND	22–25(S)	24–25(S)
*P. aeruginosa* PAO1	ND	ND	33–35(S)	ND	ND	ND	23–24(S)	29–30(S)

Antibiotic susceptibility results. Tests were performed using disc-diffusion plates with the results listed representing the range of diameters (mm) measured for the inhibitory zones from three independent tests. Strains showing resistance are listed in bold.

^a^The antibiotics tested were vancomycin (Van), rifampin (Rif), ciprofloxacin (Cip), clindamycin (Clin), oxacillin (Oxa), erythromycin (Ery), gentamycin (Gent) and tobramycin (Tob).

^b^Range of diameters (mm) seen for the inhibitory zones. (S) – susceptible; (R) – resistant.

^c^Not done.

**Table 4:** Minimum inhibitory concentration results.

Strain	Commercial Violacein	NI28 Violacein	Commercial Violacein	NI28 Violacein
	Mueller-Hinton Media		Modified M9 Media	
*S. aureus*				
ATCC 25923	15 μM	15 μM	1.8 μM	1.8 μM
Clinical Isolate	15 μM	15 μM	0.9 μM	1.8 μM
CCARM 0201	15 μM	15 μM	1.8 μM	1.8 μM
CCARM 3090	15 μM	15 μM	1.8 μM	1.8 μM
CCARM 3840	15 μM	15 μM	0.9 μM	1.8 μM
*K. pneumoniae* ATCC 13883	>30 μM	>30 μM	>30 μM	>30 μM
*P. aeruginosa* PAO1	>30 μM	>30 μM	>30 μM	>30 μM

The concentration listed is that which showed no growth after 24 hours in the resazurin microplate tests using the modified M9 media. The highest crude violacein concentration tested was 60 μM.
